# Adherence to Herpes Zoster (Shingles) Catch-Up Campaign at the Romagna Local Health Authority (Italy), a Multi-Center Retrospective Observational Study

**DOI:** 10.3390/vaccines10101770

**Published:** 2022-10-21

**Authors:** Andrea Ceccarelli, Susan Scrimaglia, Virginia Fossi, Luigi Ceccaroni, Andrea Federici, Chiara Reali, Raffaella Angelini, Giulia Silvestrini, Francesco Sintoni, Maria Pia Fantini, Davide Gori, Francesca Righi, Marco Montalti

**Affiliations:** 1 Operative Unit of Hygiene and Public Health-Cesena, Department of Public Health, Romagna Local Health Authority, 47522 Cesena, Italy; 2 Hygiene Unit, Department of Biomedical and Neuromotor Sciences, University of Bologna, 40126 Bologna, Italy; 3 Operative Unit of Hygiene and Public Health-Ravenna, Department of Public Health, Romagna Local Health Authority, 48121 Ravenna, Italy; 4 Cesena and Rubicone’s Health District, Romagna Local Health Authority, 47522 Cesena, Italy

**Keywords:** Herpes Zoster, shingles, catch-up campaign, Zostavax, Shingrix, vaccine hesitancy, vaccine uptake

## Abstract

Herpes Zoster (shingles) is an infection that occurs when varicella-zoster virus reactivates from the latent state. Incidence and severity of Herpes Zoster disease increase with age. Antiviral drugs are the elective treatment; however, prevention of disease reactivation through effective and safe vaccines is available in Italy out-of-pocket from age 65 onwards. The Romagna Local Health Authority (northern Italy) administered catch-up vaccinations in March–May 2022 for immunizations not performed during the COVID-19 pandemic. In this study, adherence rates to the catch-up campaign and recall activities adopted in two centers were investigated. The uptakes for only the catch-up vaccinations were 11.4% and 12.4%. Having suffered from Herpes Zoster or having family members who suffered from it would not seem to be drivers of increased uptake. Although sending text-messages to all involved patients was the main motivation for vaccine uptake (85.7–95.1%), word of mouth and web/news advertising also contributed to adoption in Center No. 2. In both centers, the need for greater synergy between public health departments and general practitioners to engage their patients emerged, as did the need for additional recall measures. Studying the main drivers of vaccine hesitancy, especially at the local level, can help in targeting campaigns and catch-up activities in order to achieve widespread acceptance.

## 1. Introduction

Herpes Zoster (HZ) is a disease caused by the reactivation of the varicella zoster virus (VZV) previously contracted, which is capable of remaining inactive in the nervous tissue for years. Direct contact with shingle blisters can spread VZV to people who have never had chickenpox or have never been vaccinated for chickenpox. Reactivation of VZV might be associated with a sudden lowering of the immune defenses, periods of strong psychophysical stress, immunosuppressive drug therapies (i.e., transplants, dialysis, tumors, etc.), diseases that alter or compromise the immune system, and immunosenescence. Although HZ can occur at any age, the incidence increases with age, with an estimated lifetime risk of 1 in 4 [1].

In temperate climates data show that in people not covered from any varicella vaccination program, the lifetime risk of VZV infection is greater than 95% [2], with an annual HZ incidence rate of more than 50,000 cases in people over the age of 70, data from General Practitioner (GP)-based studies in the immunocompetent population in England and Wales [3].

The incidence and severity of VZV disease increase with age, leading in some cases to serious complications such as PostHerpetic Neuralgia (PHN) as well as other neurological sequelae, ophthalmic zoster with facial palsy due to the involvement of the eye-associated dermatome, retinitis, optic neuritis [4], and systemic disease for reactivation of the virus and dissemination into the dermatome’s field (lungs, liver, gut, and brain, leading to pneumonia, hepatitis, encephalitis, and disseminated intravascular coagulopathy).

PHN rates by age cohorts in immunocompetent people found in a study by Van Hoek et al. in the UK showed an increasing trend from nine percent in the age stratum 60–64 years up to fifty-two percent over the age of 85. These rates increase if one considers people with impaired immune systems [3].

Two licensed HZ vaccines are currently available in Italy: Zostavax^®^ contains a live attenuated virus derived from the Oka/Merck strain of VZV [5], and Shingrix^®^ is a recombinant vaccine and contains VZV glycoprotein E antigen produced by recombinant DNA technology, adjuvanted with AS01 [6]. As of March 2022, the Romagna Local Health Authority (LHA) started a catch-up HZ vaccination campaign for cohorts born between 1955 and 1957, together with the usual annual vaccination offered to people aged 65 or over, as mentioned by the 2017 National Immunization Plan (NIP) [7]. Every year in Italy, HZ vaccination is offered actively to people aged 65 (born from 1952 onwards, first cohort involved), and patients have the right to get the vaccine out-of-pocket even in the following years.

Catch-up vaccinations are defined as actions carried out in order to vaccinate an individual who, for any reason, has not received vaccines for their eligibility criteria as defined by the national immunization schedule [8]. Considering that the COVID-19 pandemic had an impact on healthcare service assets, high quality supplementary immunization activities and catch-up programs are needed in order to fill the gaps, thus impacting on vaccine coverage across the globe [9]. Together with the detrimental effect of the pandemic on all the other vaccination programs other than COVID-19 another issue to deal with is vaccine hesitancy (VH). VH is defined by the WHO Scientific Advisory Group of Experts (SAGE) working group as a “delay in acceptance or refusal of vaccination despite the availability of vaccination services” [10]. VH includes not only outright refusals to vaccinate, but also the whole spectrum of negative attitudes toward vaccination, such as reluctant acceptance [11].

Measuring vaccine-specific hesitancy at the local level, as well as understanding the attitudes and beliefs that underpin it, is required to combat VH and thus target public health strategies and interventions [12]. The aims of the current study were to study the HZ catch-up vaccination campaign adherence in the Romagna LHA (northern Italy) and to observe the effectiveness of the implemented catch-up activities.

## 2. Materials and Methods

This multi-center retrospective observational study was conducted in two of the four units of the Public Health Department (PHD) of an Italian LHA conducting an HZ catch-up vaccination campaign in March–May 2022. The two centers of the study covered about half of the Romagna LHA, with a territory of about five thousand square kilometers and a population of 1,124,896 residents.

### 2.1. Study Population

The HZ catch-up vaccination campaign was aimed at the cohorts of those born in 1955, 1956, and 1957 (only in one center), assisted by the Romagna LHA, and who had not previously been vaccinated for HZ. These cohorts were chosen due to missed targeted calls by the LHA during the COVID-19 pandemic.

### 2.2. Study Setting

In the two centers involved in the study (Cesena and Ravenna), different timeframes and design for the HZ catch-up vaccination campaign were used:

– Cesena (center No. 1): at the beginning of March 2022 (approximately 20 days before the start of the vaccination campaign), text-messages were sent to the cohorts involved in the campaign (n = 2410 born in 1955; n = 2631 in 1956; and n = 2709 in 1957). At the beginning of the month, informative emails were also sent by the PhD personnel to GPs. Finally, a press/online advertising campaign was conducted approximately one week before the start of the campaign. Once started, the campaign lasted two weeks (21–31 March 2022);
– Ravenna (center No. 2): at the beginning of March 2022 (approximately 30 days before the start of the vaccination campaign), text-messages were sent to the cohorts involved in the campaign (n = 2417 born in 1955 and n = 2487 in 1956). Text-messages to those born in 1957 were scheduled to be sent the following fall. At the beginning of March 2022, informative emails were also sent by the PhD personnel to GPs. Finally, a press/online advertising campaign was conducted approximately two weeks before the start of the campaign. Once started, the campaign lasted six weeks (6 April–20 May 2022).

In both centers, before proceeding with the vaccination, each individual attending the vaccination center underwent an interview with a medical doctor (or other PhD personnel) to collect their vaccination and anamnestic history.

### 2.3. Data Extraction

Data was extracted anonymously from each vaccination history record chart by six independent reviewers (A.C., S.S., V.F., L.C., A.F., and M.M.) in May 2022. Disagreement on extracted data was discussed with an independent tie breaker (F.R.). Variables extracted from each chart were: “Type of HZ vaccine”, “Gender”, “Age”, “Neurological disorders”, “Primary immunodeficiency”, “Allergies”, “Vaccine allergies”, “Iatrogenic immunosuppression”, “Personal history of HZ”, “Relatives’ history of HZ, and “Source of information used to find out about the catch-up vaccination campaign”. Personal and family history of HZ were included because they are thought to be motivators for vaccine acceptance or refusal. Data were collected in Microsoft Excel (Microsoft Corporation).

### 2.4. Statistical Analysis

Variables were described as absolute frequencies and percentages. The determinants of use of a specific source of information to find out about the main catch-up activity adopted (text-message) were assessed by a multivariate analysis. Results of multivariate analyses are presented as odds ratios (ORs) with standard error (SE) and a 95% confidence interval (CI). A backward stepwise analysis was carried out in order to define the variables to be included in the final multiple logistic regression model, according to the principles of parsimony and biological plausibility. The statistical significance level was set up at *p* < 0.05. All analyses were carried out using Stata Statistical Software 15 (StataCorp, College Station, TX, USA).

## 3. Results

### 3.1. Main Sample Features

In total, 11.4% of the campaign’s targets in center No. 1 (880 out of 7750 people invited) and 12.4% of the campaign’s targets in center No. 2 (609 out of 4904) were vaccinated during the catch-up campaign.

In both centers, the live attenuated virus vaccine (*n* = 836; 95.0% in center No. 1 and *n* = 592; 97.2% in center No. 2) was the most frequently injected vaccine. The studied population consisted of 50.9% males for center No. 1 and 46.1% for center No. 2.

Regarding pathological anamnesis, the most frequent variable was “allergies” (*n* = 76; 8.7% in center No. 1 and *n* = 86; 14.1% in center No. 2). As for the variable of “History of Herpes Zoster”, 217 (33.3%) and 201 (37.4%) reported having some relatives who had the infection, and 114 (17.4%) and 91 (17.0%) have personally reported the infection, respectively in each center.

The main sources of information were text-messages (*n* = 621; 95.1%, and *n* = 456; 85.7%). Detailed characteristics are summarized in Table 1.

### 3.2. Multiple Regression Analysis

A regression model was used to determine variables associated with the source of information. Results showed that center No. 2 (OR: 0.31, SE: 0.09, *p* < 0.001, 95% C.I.: 0.18–0.53) was associated with a reduction in the likelihood of the use of text-messages as a warning for vaccination. Results are summarized in Table 2.

## 4. Discussion

In this multi-center study, an adherence rate of 11.4–12.4% to the HZ catch-up campaign was found in Romagna LHA, Italy. Although the most impactful catch-up activity adopted was clearly sending text-messages to every single recipient of the campaign’s targeted cohorts, nearly 15% in center No. 2 reported adherence thanks to other catch-up activities. The different timeframes and designs used in the two centers where the study was performed did not lead to major differences in uptake.

Vaccine uptake rates for HZ are not currently being tracked by the Ministry of Health in Italy, despite the fact that the vaccination is included in the NIP and a 50% coverage rate has been identified as a target for 2020. Available data on VH for HZ vaccination are limited in the scientific literature but suggests that VH rates for this vaccine are higher than other vaccines such as COVID-19 and flu [13,14]. According to studies conducted in Italy in 2018 and in the United States in 2017, acceptance rates for HZ were around 20.7–22.6% and 17.8–46.6%, respectively [15,16], while even lower rates were reported in settings where the vaccine was not offered out-of-pocket [17]. In another recent study in the UK, the uptake rate for HZ found after a three-year extensive vaccination program was 58–72% among the targeted cohorts [18]. Given the UK study, the coverage rates achieved by our catch-up campaign might seem low. However, to get a more complete picture of our LHA annual campaign, coverage rates achieved by the ongoing routine HZ campaign should also be considered. Other possible reasons why higher rates have been achieved in the UK include: more cohorts were considered in their programme than in our LHA’s catch-up campaign; and the UK cohorts had three years to decide whether or not to get the out-of-pocket vaccine.

HZ incidence, similar worldwide, shows no seasonal or epidemic pattern and correlates with population age. About one out of four individuals experiences HZ reactivation during their lifetime [19,20], and, as the elderly and frail population increases, an increase in HZ cases is expected in the near future [21]. The reactivation rate is consistent with findings in our study about people who reported previous HZ infection or had relatives who had been infected. For this reason, a previous HZ infection would not seem to be a significant driver for vaccine uptake. However, a previous study conducted in the UK showed that HZ VH was associated with perceived barriers, perceived control of the disease, and a previous history of shingles [22]. This finding could be explained by a low awareness and knowledge of the vaccine efficacy, and, in order to counteract this specific health illiteracy, media coverage and promotion campaigns held by local PhD personnel could be implemented.

In our catch-up campaign, text-messages were the main tool used, and it has been well demonstrated how they are an excellent means of increasing vaccination rates [23,24,25]. However, the low adherence rate found in our study suggests that text-message invitations alone could not be enough in our local context. Co-administration of vaccines is among other useful tools for achieving good vaccine coverage. As occurred in the Calabria Region (Italy), the adherence rates were higher than ever after the co-administration of HZ and pneumoccal vaccine (PCV13 vaccine) was introduced [15]. Similar co-administrations were tried with COVID-19 and flu vaccines [26,27] and could be carried out with tetanus vaccine or during scheduled family visits for newborns (so-called cocoon vaccinations).

Other factors contributing to higher vaccination rates include GPs, as they have a vital role in recommending vaccination to eligible patients [28]. Moreover, their advice may have a positive influence on patients’ willingness to be vaccinated. Although in our study, the number of recipients who get the HZ vaccine following their GPs’ suggestion is small, GPs have an overview of the patients’ diseases and medication and might be able to persuade them to accept the vaccination. In a Dutch study [29] in which GPs offered HZ vaccination and annual flu vaccination to their patients at the same time, only 690 patients (39%) accepted the shingles vaccination, compared with 1349 patients (76%) for the flu vaccination. The determinants of HZ vaccination refusal were: perceived lack of recommendation by the GP, unwillingness to follow the doctor’s advice, perceived low risk of contracting HZ, perceived short duration of HZ pain, and the idea that vaccinations weaken natural defenses. Therefore, it would be necessary to improve the knowledge of both GPs, encouraging their participation in educational events on vaccination and of patients, regarding the HZ disease and how to prevent it [30].

As a result, several factors could potentially improve adult vaccination coverage, including sending automatic reminders for patients, implementing public education campaigns, offering the vaccine in co-administration with other vaccines, and encouraging GPs to recommend vaccination to eligible patients [31].

This study has some limitations that must be considered. First of all, this catch-up campaign was carried out during the COVID-19 mass vaccination campaign across Italy, leading to a possible lower adherence to the HZ campaign. The two centers carried out two different methods of the catch-up campaign management. In particular, center No. 2 did not contact the cohort of those born in 1957 by postponing the text-message to September 2022. Pathological anamnesis and history of HZ infection were self-reported (not checked with previous medical charts) and might be subject to reporting bias. The PhD personnel who collected the medical history were numerous and different between the two centers. However, there were only six vaccination history record chart reviewers to ensure uniformity in DB creation. Finally, vaccination adherence to the catch-up campaign alone was analyzed, whereas to calculate overall vaccination adherence it would have been necessary to also study adherence to the ordinary ongoing campaign.

## 5. Conclusions

Our results contributed to the study of adherence to a catch-up campaign conducted at a local level with a large sample size and on a bicenter basis. Although text-messages have made an excellent contribution in fighting VH, the need for greater synergy between PhD personnel and GPs has emerged. The latter could be more extensively involved in calling their patients for shingles vaccination. Earlier publicizing of vaccination campaigns through the web and other media at the local level could also help to increase adherence rates. Different geographical and cultural contexts play an important role and acting locally can be the turning point for effective and efficient strategies. This study can be an important piece of knowledge to strengthen the need for catch-up activities at different levels of community health care.

## Figures and Tables

**Table 1:** Main sample features (*n* = 1489).

		Center	
		No. 1 (*n* = 880)	No. 2 (*n* = 609)
**Vaccine**	Live attenuated virus	836 (95.0)	592 (97.2)
	Recombinant vaccine	44 (5.0)	17 (2.8)
**Gender**	Male	448 (50.9)	281 (46.1)
	Female	432 (49.1)	328 (53.9)
**Age**	67	278 (31.6)	303 (49.8)
	66	286 (32.5)	278 (45.7)
	65	299 (34.0)	24 (3.9)
	Other	17 (1.9)	4 (0.7)
**Pathological Anamnesis**	Neurological disorders	18 (2.1)	9 (1.5)
	Primary immunodeficiency	13 (1.5)	12 (2.0)
	Allergies	76 (8.7)	86 (14.1)
	Vaccine allergies	6 (0.7)	3 (0.5)
	Iatrogenic immunosuppression	32 (3.6)	11 (1.8)
**History of Herpes Zoster**	Personal	114 (17.4)	91 (17.0)
	Relatives	217 (33.3)	201 (37.4)
**Vaccination Campaign-source of information**	Text-message	621 (95.1)	456 (85.7)
	General practitioner	10 (1.5)	6 (1.1)
	Family/friends	14 (2.1)	48 (9.0)
	Web	1 (0.2)	7 (1.3)
	News	3 (0.5)	4 (0.8)
	Other	4 (0.6)	11 (2.1)

**Table 2:** Variables associated with the source of information used in a multiple regression analysis.

Use of Text-Messages as a Warning for the Vaccination Campaign		OR	SE	*p*-Value	95% C.I.
Center	Center No. 1	1			
	Center No. 2	0.31	0.09	**<0.001**	0.18–0.53
Gender	Male	1			
	Female	0.91	0.22	0.704	0.58–1.45
Age	65	1			
	66	0.88	0.21	0.587	0.56–1.39

Note: All the listed variables were included in the model. Vaccinated individuals not belonging to the cohorts targeted by the catch-up campaign were excluded (21 out of 1489, 1.4%). Patients born in 1957 were excluded because they were targeted by only one center (No. 1) (323 out of 1489, 21.7%).

## Data Availability

The data presented in this study are available on request from the corresponding author.
